# Supplementary material for: Pregnancy-specific responses to COVID-19 revealed by high-throughput proteomics of human plasma
Source: Commun Med (Lond). 2023 Apr 4;3:48. doi: 10.1038/s43856-023-00268-y (PMC10071476; doi:10.1038/s43856-023-00268-y)
Supplement: Supplementary file 12 — Supplementary Information [file 43856_2023_268_MOESM12_ESM.pdf]

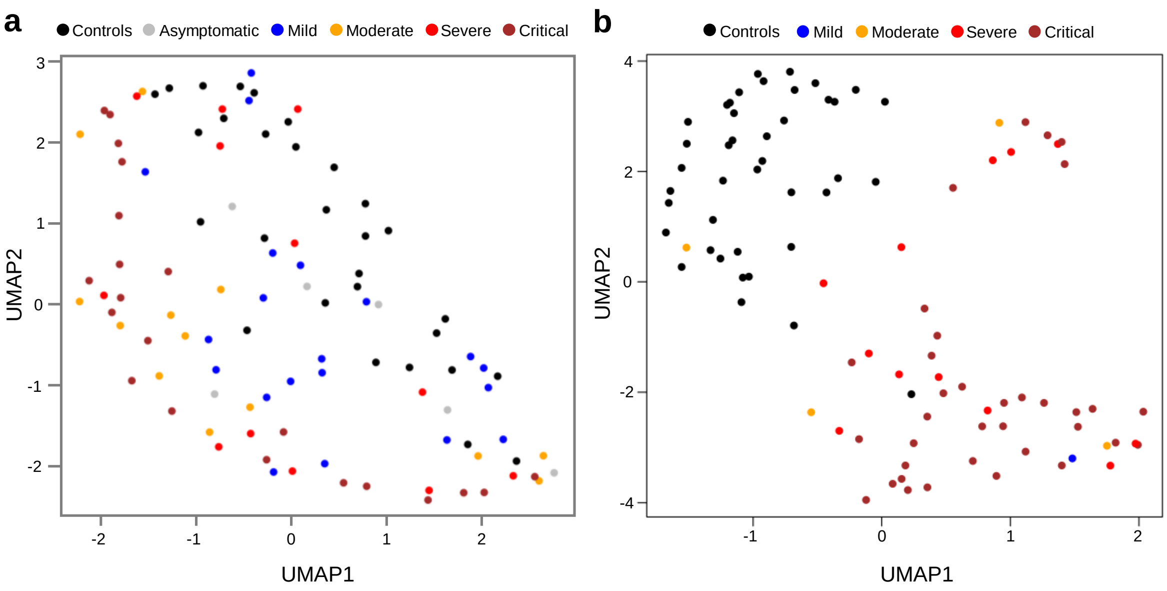

Supplementary Fig. 1. UMAP plots representing the proteomic profiles in **(a)** pregnant and **(b)** non-pregnant patients by disease severity. Black = control, grey = asymptomatic case, blue = mild case, yellow = moderate case, red = severe case, brown = critical case.

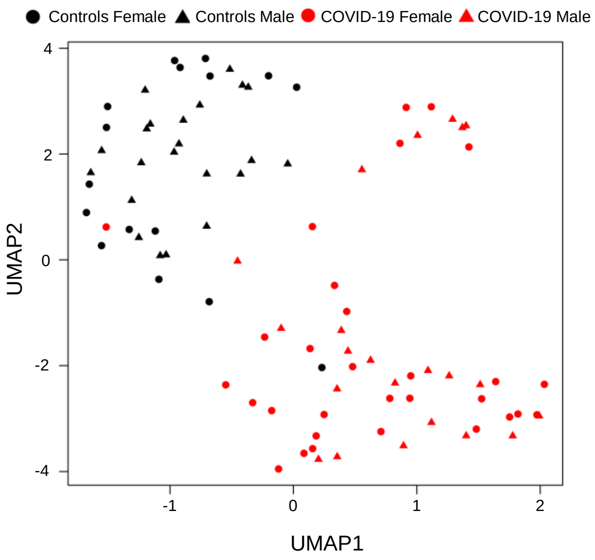

Supplementary Fig. 2. UMAP plot representing the proteomic profiles of non-pregnant patients according to sex. Black = control, red = case. Circle = female, triangle = male.

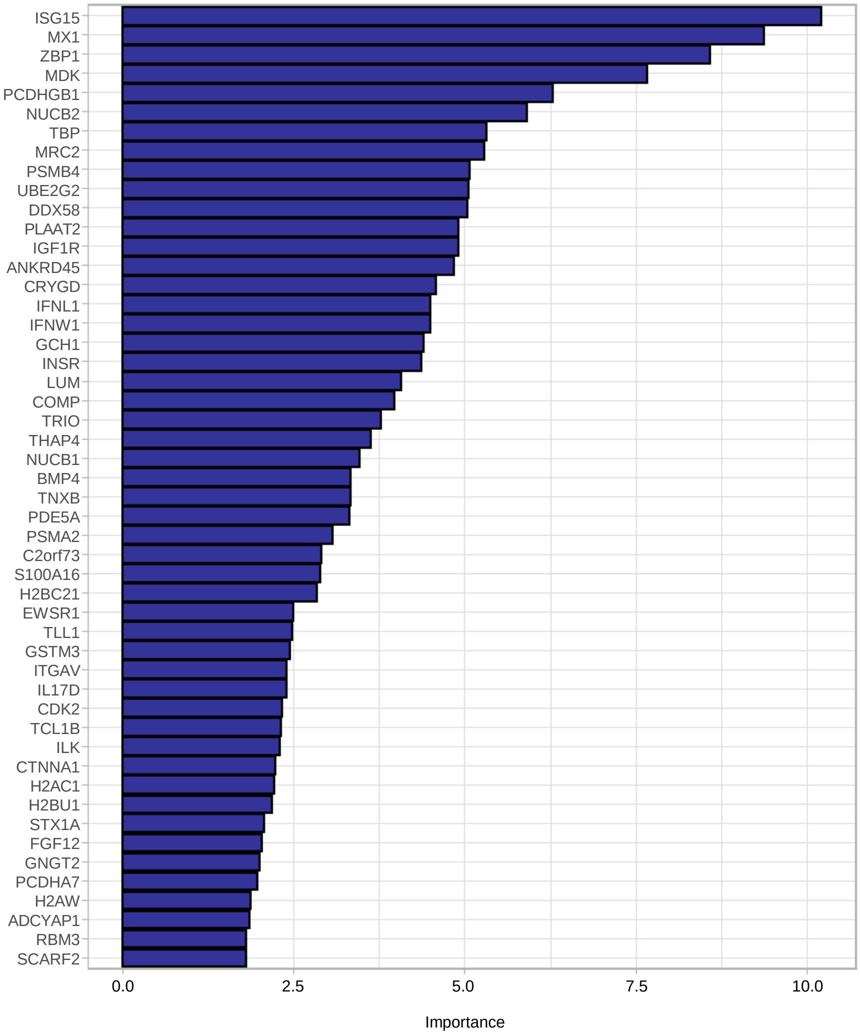

Supplementary Fig. 3. Bar plot displaying the relative importance of the top 50 proteomic predictors for distinguishing moderate COVID-19 cases from controls.

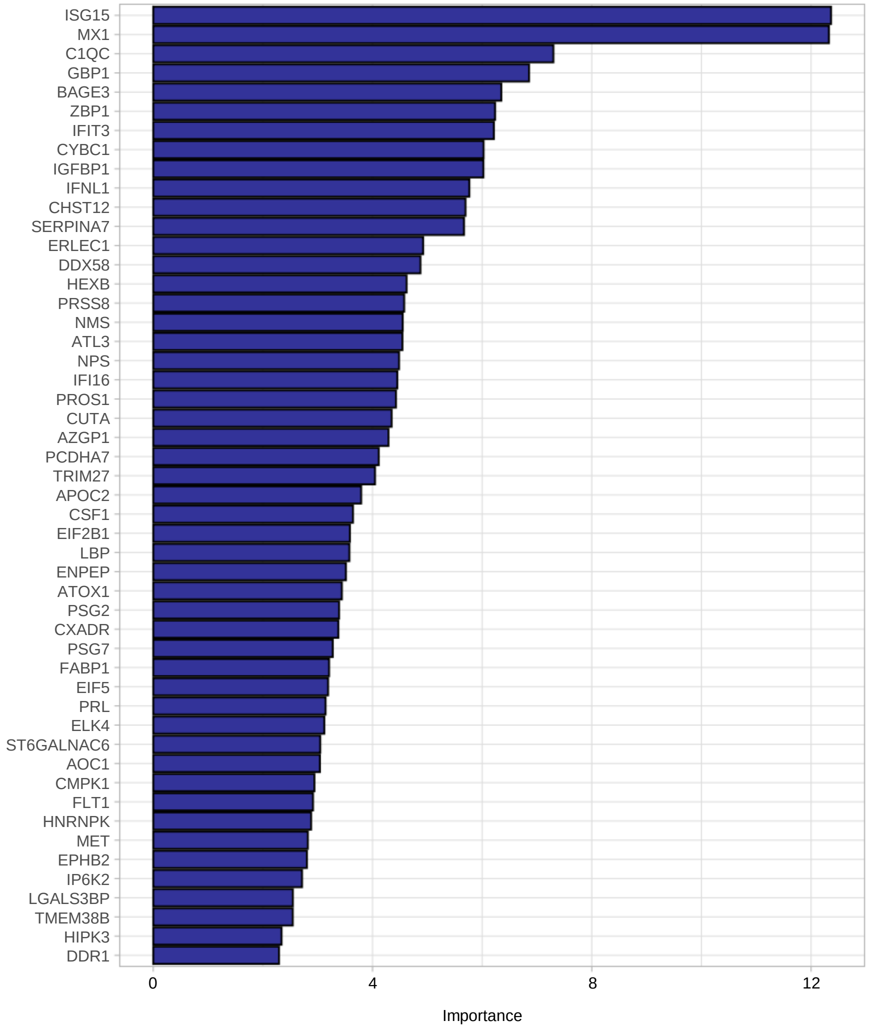

Supplementary Fig. 4. Bar plot displaying the relative importance of the top 50 proteomic predictors for distinguishing asymptomatic/mild COVID-19 cases from controls.
