# Supplementary material for: Pregnancy-specific responses to COVID-19 revealed by high-throughput proteomics of human plasma
Source: Commun Med (Lond). 2023 Apr 4;3:48. doi: 10.1038/s43856-023-00268-y (PMC10071476; doi:10.1038/s43856-023-00268-y)
Supplement: Supplementary file 13 — Description of Additional Supplementary Files [file 43856_2023_268_MOESM13_ESM.pdf]

Description of Additional Supplementary Files

File Name: Supplementary Data 1

Description: Proteins differentially regulated with COVID-19 in non-pregnant patients compared to non-pregnant controls.

File Name: Supplementary Data 2

Description: Clustering of 486 proteins with shared perturbation in pregnant and non-pregnant women with COVID-19.

File Name: Supplementary Data 3

Description: Proteins differentially regulated by COVID-19 in pregnant women compared to non-infected pregnant controls.

File Name: Supplementary Data 4

Description: Biological processes enriched among differentially regulated proteins in pregnant women with COVID-19.

File Name: Supplementary Data 5

Description: Biological processes enriched among differentially regulated proteins in non-pregnant COVID-19 patients.

File Name: Supplementary Data 6

Description: Biological processes enriched among differentially regulated proteins in pregnant and non-pregnant women with COVID-19 according to disease severity. 1 = significant enrichment ($q < 0.1$).

File Name: Supplementary Data 7

Description: C2 pathways enriched among differentially regulated proteins in pregnant COVID-19 patients.

File Name: Supplementary Data 8

Description: C2 pathways enriched among differentially regulated proteins in non-pregnant COVID-19 patients.

File Name: Supplementary Data 9

Description: C2 pathways enriched among differentially regulated proteins in pregnant and non-pregnant women with COVID-19 according to disease severity. 1 = significant enrichment ($q < 0.1$).

File Name: Supplementary Data 10

Description: Proteins differentially regulated in opposite directions between pregnant and non-pregnant COVID-19 patients.

File Name: Supplementary Data 11

Description: Biological processes and C2 pathways enriched among proteins regulated in opposite directions between pregnant and non-pregnant COVID-19 patients.
