# Supplementary material for: Pregnancy-specific responses to COVID-19 revealed by high-throughput proteomics of human plasma
Source: Commun Med (Lond). 2023 Apr 4;3:48. doi: 10.1038/s43856-023-00268-y (PMC10071476; doi:10.1038/s43856-023-00268-y)
Supplement: Supplementary file 14 — Reporting Summary [file 43856_2023_268_MOESM14_ESM.pdf]

nature portfolio

Corresponding author(s): Nardhy Gomez-Lopez, Adi L. Tarca, Roberto Romero

Last updated by author(s): Feb 15, 2023

Reporting Summary

Nature Portfolio wishes to improve the reproducibility of the work that we publish. This form provides structure for consistency and transparency in reporting. For further information on Nature Portfolio policies, see our Editorial Policies and the Editorial Policy Checklist.

Statistics

For all statistical analyses, confirm that the following items are present in the figure legend, table legend, main text, or Methods section.

n/a	Confirmed	
☐	☒	The exact sample size (*n*) for each experimental group/condition, given as a discrete number and unit of measurement
☐	☒	A statement on whether measurements were taken from distinct samples or whether the same sample was measured repeatedly
☐	☒	The statistical test(s) used AND whether they are one- or two-sided<br>*Only common tests should be described solely by name; describe more complex techniques in the Methods section.*
☐	☒	A description of all covariates tested
☐	☒	A description of any assumptions or corrections, such as tests of normality and adjustment for multiple comparisons
☐	☒	A full description of the statistical parameters including central tendency (e.g. means) or other basic estimates (e.g. regression coefficient) AND variation (e.g. standard deviation) or associated estimates of uncertainty (e.g. confidence intervals)
☐	☒	For null hypothesis testing, the test statistic (e.g. *F*, *t*, *r*) with confidence intervals, effect sizes, degrees of freedom and *P* value noted<br>*Give P values as exact values whenever suitable.*
☒	☐	For Bayesian analysis, information on the choice of priors and Markov chain Monte Carlo settings
☐	☒	For hierarchical and complex designs, identification of the appropriate level for tests and full reporting of outcomes
☐	☒	Estimates of effect sizes (e.g. Cohen's *d*, Pearson's *r*), indicating how they were calculated

Our web collection on statistics for biologists contains articles on many of the points above.

Software and code

Policy information about availability of computer code

Data collection	Proteomic data was obtained from Somalogic Inc, using the SOMAScan v4.1 platfrom.
Data analysis	Open source R packages were used for analysis and described in the Methods section.

For manuscripts utilizing custom algorithms or software that are central to the research but not yet described in published literature, software must be made available to editors and reviewers. We strongly encourage code deposition in a community repository (e.g. GitHub). See the Nature Portfolio guidelines for submitting code & software for further information.

Data

Policy information about availability of data

All manuscripts must include a data availability statement. This statement should provide the following information, where applicable:

- Accession codes, unique identifiers, or web links for publicly available datasets
- A description of any restrictions on data availability
- For clinical datasets or third party data, please ensure that the statement adheres to our policy

The majority of the data generated in this study are included in the manuscript and/or in the Supplementary Materials. Proteomic data generated in this study are available at the Gene Expression Omnibus (accession number GSE207015). Reviewer access token: onctumuexhkfvod

Human research participants

Policy information about [studies involving human research participants and Sex and Gender in Research.]

Reporting on sex and gender	Patient sex information was available and considered in the analysis of data from the non-pregnant population.
Population characteristics	Population characteristics including sex, age, bmi were provided part of the Gene Expression Omnibus submission. (GSE207015).
Recruitment	Pregnant patients were enrolled at admission for labor and delivery or at the time of attending the clinic for any obstetrical indications. All patients were screened for Covid 19 according to the standard clinical care. Of all controls (no Covid19) those who provided samples at the same gestational age window as the cases were retained. Analyses of data of pregnant patients included adjustment for gestational age at sampling, BMI, and maternal age. Non-pregnant patients were enrolled at the time of admission for any medical reasons and they were all tested for COVID-19. Analyses of non-pregnant individuals included accounting for age and sex of participants as well as chronic hypertension status, as described in the methods section.
Ethics oversight	Biomedical Research Ethics Committee of the Fundacion Valle del Lili (Protocol No. 1611)

Note that full information on the approval of the study protocol must also be provided in the manuscript.

Field-specific reporting

Please select the one below that is the best fit for your research. If you are not sure, read the appropriate sections before making your selection.

☒ Life sciences ☐ Behavioural & social sciences ☐ Ecological, evolutionary & environmental sciences

For a reference copy of the document with all sections, see nature.com/documents/nr-reporting-summary-flat.pdf

Life sciences study design

All studies must disclose on these points even when the disclosure is negative.

Sample size	Sample size was dictated by patient/sample availability.
Data exclusions	No samples were excluded from the analysis after generation of proteomics data.
Replication	No technical replication was involved, but only biologic replication.
Randomization	Samples were randomized and processed in batches.
Blinding	The proteomic data was generated by SomaLogic Inc without access to patient group information.

Reporting for specific materials, systems and methods

We require information from authors about some types of materials, experimental systems and methods used in many studies. Here, indicate whether each material, system or method listed is relevant to your study. If you are not sure if a list item applies to your research, read the appropriate section before selecting a response.

Materials & experimental systems

n/a	Involved in the study	
☒	☐	Antibodies
☒	☐	Eukaryotic cell lines
☒	☐	Palaeontology and archaeology
☒	☐	Animals and other organisms
☒	☐	Clinical data
☒	☐	Dual use research of concern

Methods

n/a	Involved in the study	
☒	☐	ChIP-seq
☒	☐	Flow cytometry
☒	☐	MRI-based neuroimaging
